# Determination of the Gender-Related Differences on Native Femoral Anatomy Using Three-Dimensional Computerized Tomography Models in Caucasian Population

**DOI:** 10.7759/cureus.16235

**Published:** 2021-07-07

**Authors:** Oğuzhan Tanoğlu

**Affiliations:** 1 Orthopedics and Traumatology, Erzincan Binali Yıldırım University, Erzincan, TUR

**Keywords:** computer generated three-dimensional imaging, anteversion, retroversion, caucasian, computer-assisted image processing

## Abstract

Introduction

Three-dimensional (3D) modelling, which has an increasing interest in the literature, could help surgeons to better understand the lesions by visualizing the real anatomical features compared to plain radiographs and two-dimensional (2D) tomography images. We aimed to evaluate the native femoral anatomical features of Turkish females and males using 3D computed tomography models.

Methods

We evaluated the right femoral anatomical features of 60 females and 60 males between 31 and 65 years of age creating 3D computerized tomography models. The gender-specific differences of femoral neck inclination and anteversion, femoral mechanical-anatomical axis, anatomical and mechanical lateral distal femoral, medial and lateral proximal femoral angles were measured on 3D femoral anatomical models.

Results

The mean age of our study groups was 50.6 ± 8.5. We determined a statistically significant difference between gender groups in terms of mean femoral neck anteversion angles (p = 0.009). We observed the retroversion of the femoral neck in 12 adults (10%). The mean values of femoral neck inclination, femoral mechanical - anatomical angle, anatomical and mechanical lateral distal femoral angles, medial and lateral proximal femoral angles did not differ any statistical significance between gender groups.

Conclusion

Although the anatomical angle measurements except femoral neck anteversion, did not differ significantly between gender groups of our study, there were differences between mean anatomical angles when compared to other studies in the literature, which investigate the different races or Caucasian population. Through 3D anatomical data, more compatible implants, prosthesis or biomaterials can be produced by determining gender and race-specific anatomical differences.

## Introduction

It is important to understand the native anatomical and biomechanical features of the bones and joints to achieve the successful results in Orthopedics and Trauma surgery [1-4]. Therefore, over time the different imaging methods have been developed for detailed investigation of the native anatomical and morphological changes. Computed tomography is a commonly used imaging technique for the diagnosis of bone lesions and the preoperative planning in deformity, arthroplasty, trauma and spine surgeries [1,3,5,6]. Nowadays, the two-dimensional (2D) tomography images could be converted into the three-dimensional (3D) models using specialized imaging programs. 3D modelling, which have an increasing interest in the literature, that could help the surgeons to better understand the lesions by visualizing the real anatomical features compared to plain radiographs and 2D tomography images [7-9].

Gender effects on native femoral anatomy is especially important for the development of suitable implants and prosthesis. Implant modifications and optimizations, which are made in accordance with gender-specific anatomical differences, could increase the postoperative functional results [7]. The effects of gender, age and race on anatomical features using the tomographic measurements, were largely investigated in the literature [1,3,4,8-10]. In some of these studies, the femoral markings and measurements were performed using 2D methods [3,4], while in a limited number of studies all the femoral markings and measurements were performed on 3D models [1,8-10]. Despite these publications, the complex 3D anatomy of the femur is still not fully defined [11]. 3D anatomical models are not affected by the patient's position in tomography device and the anatomical landmarks could be more easily identified with manual or autonomous methods [2,9]. Therefore, recent studies have emphasized the superiority of 3D evaluation to eliminate the difficulties in understanding the complex femoral anatomy [2,9,11].

We aimed in our current study to identify the detailed native femoral anatomical features of healthy female and male adults in the Turkish population using 3D computed tomography models.

## Materials and methods

Approval for this retrospective study was granted by the Institutional Review Board of Ankara City Hospital with the registration number E1-20-1309. The computed tomography images of the adults (60 females and 60 males) between 31 and 65 years of age, who were originated from Caucasian descent and were consulted in Orthopedics and Traumatology Department of Erzincan University between 2014 and 2019, were reviewed via the radiological records. The exclusion criteria were incomplete computed tomography images of the femur, the adults originated from different races, prior orthopedic interventions for deformity corrections, oncological interventions, hip or knee arthroplasties, fracture fixations and any radiological evidence of femoroacetabular impingement, knee or hip osteoarthritis. All the patients were anonymized with the software Mimics 21 (Materialise, Leuven, Belgium). Among the adults who met the study criteria, 60 females and 60 males were randomly selected using true random number generator program (www.random.org). Tomography scans were performed on a Siemens SOMATOM Emotion (Siemens, Erlangen, Germany) (110 kV-90 mAs, slice thickness 1.2 mm.). The right femur of the patients was selected to create a 3D anatomical model. The cortical bone of femur was pointed on 2D tomography slices and the program was automatically segmented the femoral 3D models using the advanced segment option of Mimics 21 (Materialise, Leuven, Belgium). Thresholding limits were determined as between 200-2150 Hounsfield Unit to isolate the bone tissue from medullary canal and other tissues.

**Figure 1:**
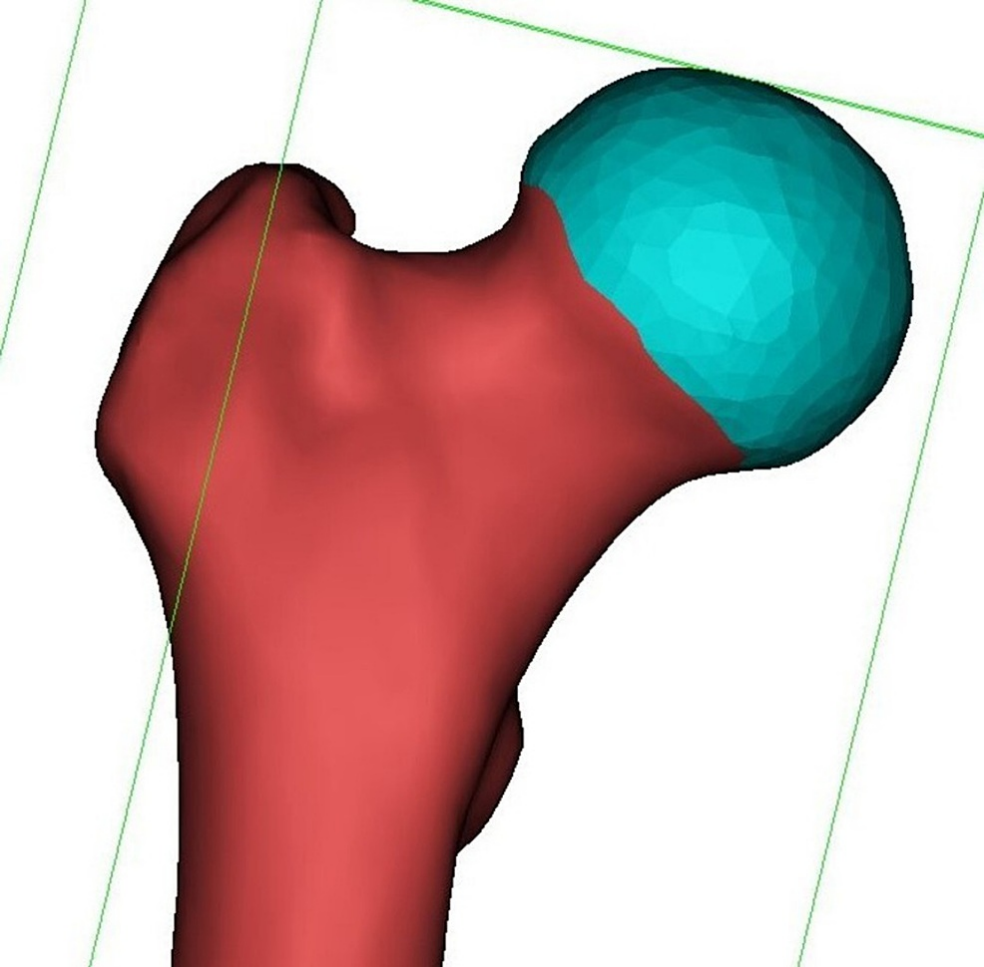
Marking of the femoral head articular surface circumferentially to create a best fit sphere.

The anatomical points were determined on the 3D femoral models using 3-Matic (Materialise, Leuven, Belgium) in accordance with previously defined methods in the literature [3,5,6,12]. The lines were drawn between the anatomical points on 3D models. The largest diameter of the femoral head was signed using the best fit diameter option. Femoral head articular surface was marked circumferentially to create a fit to surface sphere (Figure 1).

**Figure 2:**
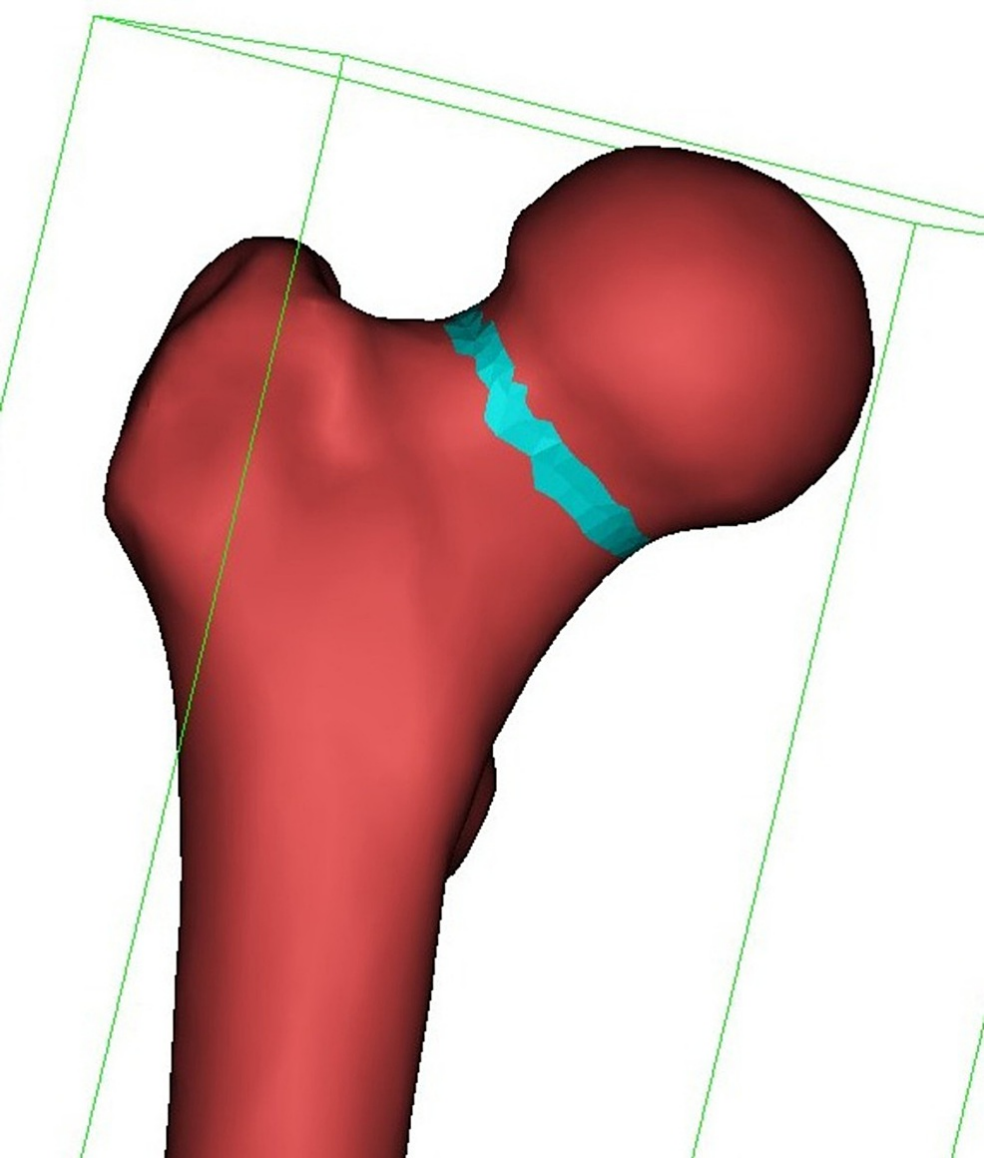
Marking of the narrowest diameter of the femoral neck circumferentially to create a best fit sphere.

The central points of femoral neck were detected using fit centerline option of computer program. The narrowest diameter of the femoral neck was detected measuring the best fit diameter option on several central points of femoral neck region. The narrowest diameter was marked circumferentially to create a fit to surface sphere (Figure 2).

**Figure 3:**
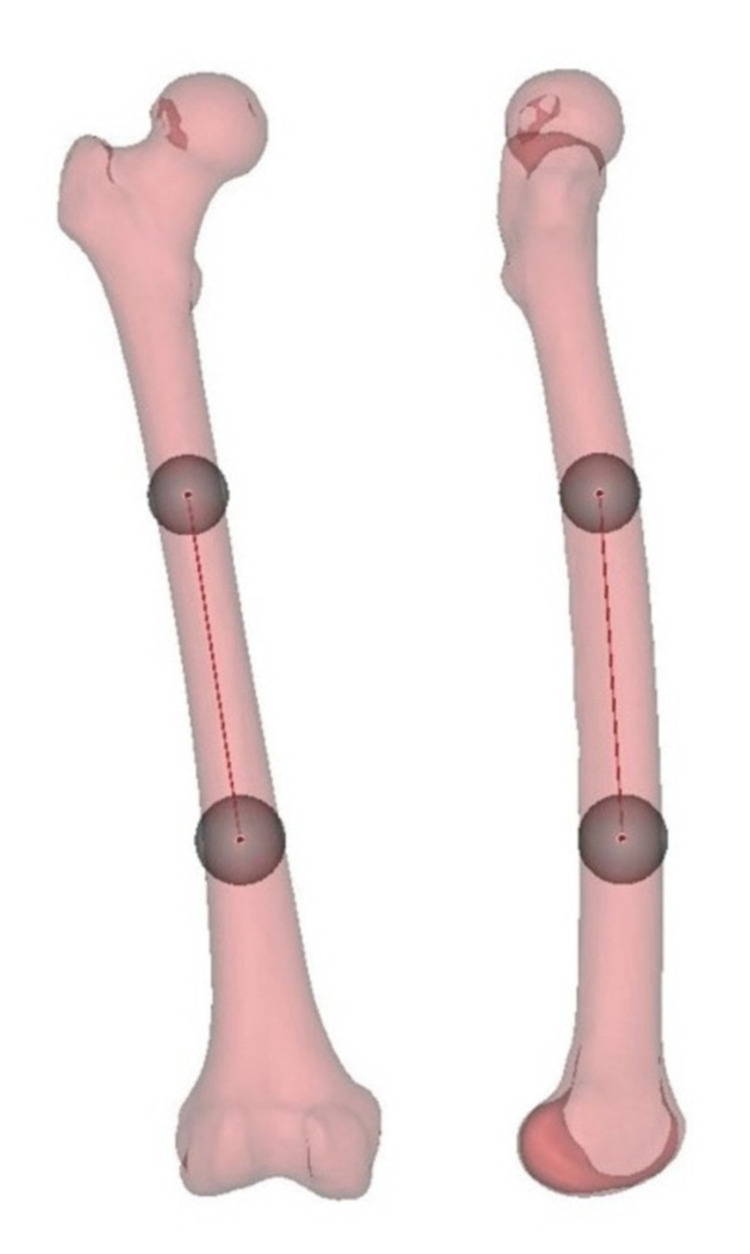
Best fit spheres on the 1/3 proximal and distal femoral shaft, anatomical axis of whole femoral model in anteroposterior view (left) and lateral view (right).

The length of the femur was measured between the tip of the trochanter major and the most superior point of the notch and longitudinally separated into three parts. The best-fit diameters of 1/3 proximal and distal femoral shaft borders were determined. The borders were marked circumferentially and two different spheres on these borders were created using the fit to surface option (Figure 3).

The definition of the anatomical points, spheres and lines on 3D models were shown in Table 1.

**Table 1:** Definition of the anatomical points, spheres and lines on 3D models.

Points	Definition
TrM	Top point of trochanter major (XZ axis)
Mepi	The most distal point of medial epicondyle (XZ axis)
Lepi	The most distal point of lateral epicondyle (XZ axis)
Mpost	The most posterior point of medial condyle (XY axis)
Lpost	The most posterior point of lateral condyle (XY axis)
Minf	The most inferior point of the medial femoral condyle (XZ axis)
Linf	The most inferior point of the lateral femoral condyle (XZ axis)
Topnotch	Top point of notch (XZ axis)
Spheres	
Headsphere	a fit to surface sphere on femoral head
Necksphere	a fit to surface sphere on the narrowest diameter of femoral neck
Proxshaft	a fit to surface sphere on the 1/3 proximal border of femoral shaft
Distshaft	a fit to surface sphere on the 1/3 distal border of femoral shaft
Lines	
TrMHead	The line between TrM and the center point of head sphere
Headneck	The line between the center points of head and neck spheres
Fanat	The line between the center points of the spheres on 1/3 proximal and distal borders of femoral shaft
Fmech	The line between the center of head sphere and Topnotch
EpiCon	The line between the most distal points of medial and lateral epicondyles
PostCon	The line between the most posterior points of medial and lateral condyles
InfCon	The line between the most inferior points of medial and lateral condyles

The 3D model was aligned with the object coordinate system of imaging program. The definition of the angles on 3D models were given in Table 2.

**Table 2:** Definition of the angles on 3D models. Headneck: The line between the center points of head and neck spheres, Fanat: femoral anatomical axis, Fmech: femoral mechanical axis, Postcon: posterior intercondylar line, InfCon: Inferior intercondylar line, TrMHead: the line between the top point of trochanter major and the center of head sphere.

Angles and axis	Definition
Femoral neck inclination (FNI)	Angle between Headneck and Fanat (XZ axis)
Femoral neck anteversion (FNA)	Angle between Headneck and PostCon (XY axis)
Femoral mechanical-anatomical axis angle (FMAA)	Angle between Fmech and Fanat (XZ axis)
Anatomical lateral distal femoral angle (aLDFA)	Angle between Fanat and InfCon (XZ axis)
Mechanical lateral distal femoral angle (mLDFA)	Angle between Fmech and InfCon (XZ axis)
Medial proximal femoral angle (MPFA)	Angle between TrMHead and Fanat (XZ axis)
Lateral proximal femoral angle (LPFA)	Angle between TrMHead and Fmech (XZ axis)

Sample size was calculated by accepting type 1 error level as 0.05 and the power of the study as 0.8. By accepting the effect size as 0.8, the total sample size was calculated to be at least 52 models using G*Power 3.1.9.7. (Düsseldorf University, Düsseldorf, Germany). Statistical analysis was performed with Statistical Package for the Social Sciences (SPSS) version 25 (2017) (IBM, NY, USA). All samples have shown normal distribution according to the Kolmogorov-Smirnov normality test. Therefore, the means and standard deviations of the samples were calculated with one way ANOVA test. The level of statistical significance was accepted as p < 0.05.

## Results

In our study, the right femur of 60 male and 60 female adults, between 31 and 65 years of age, were investigated. The mean age of the groups was 50.6 ± 8.5. The mean age and standard deviations of the groups in terms of gender were given in Table 3.

**Table 3:** The mean ± standard deviations (SD), ranges and p values of the groups. *Statistically significant according to one way ANOVA test, significance level p <0.05 FNI: femoral neck inclination; FNA: femoral neck anteversion; FMAA: femoral mechanical-anatomical angle; aLDFA: anatomical lateral distal femoral angle; mLDFA: mechanical lateral distal femoral angle; MPFA: medial proximal femoral angle; LPFA: lateral proximal femoral angle.

	Female	Male	Total	
	Mean ± SD (range)	Mean ± SD (range)	Mean ± SD (range)	p-value
Age	51 ± 10.3 (31-65)	50.3 ± 6.3 (33-58)	50.6 ± 8.5 (31-65)	0.753
FNI	125.1 ± 5.6 (114-136)	123.1 ± 6.5 (108-136)	124.1 ± 6.1 (108-136)	0.22
FNA	17.1 ± 9.9 (-5-33)	10.6 ± 8.3 (-4-29)	13.9 ± 9.6 (-5-33)	0.009*
FMAA	6.2 ± 0.8 (4-8)	6 ± 0.9 (4-7)	6.1 ± 0.8 (4-8)	0.344
aLDFA	80.6 ± 1.6 (77-85)	80.3 ± 2 (77-84)	80.4 ± 1.8 (77-85)	0.521
mLDFA	86.8 ± 1.6 (83-91)	86.3 ± 1.5 (82-89)	86.5 ± 1.6 (82-91)	0.193
MPFA	80.9 ± 4.3 (71-88)	80.8 ± 5.1 (73-98)	80.8 ± 4.7 (71-98)	0.965
LPFA	92.8 ± 4.1 (85-102)	93.1 ± 4.8 (77-100)	93 ± 4.4 (77-102)	0.814

We determined the retroversion of the femoral neck in six male adults (10%) and in six female adults (10%).

P values ​​obtained from the comparison of the groups are given in Table 3. Our study results revealed that there was a statistically significant difference between gender groups only in terms of femoral neck anteversion (FNA) angles (p = 0.009). The mean values of other angles except FNA, did not differ any statistical significance between gender groups.

## Discussion

Understanding the racial differences in the native 3D femoral anatomy is important for an accurate preoperative planning and designing more compatible orthopedic implants [1,6,7]. In the literature, there are many studies that investigate the femoral anatomical features of different populations [1-5,7,10]. Most of these studies, that analyzed the racial differences on anatomical features, have focused on the Caucasian population from western countries [2-5]; however, the studies investigating the native femoral anatomy of the populations originated from different races on 3D models are limited [7,10]. Caucasian race is a concept that defines the different communities living in a wide geographical area. We thought that there may be anatomical differences within the Caucasian race in this wide geographical area. Therefore, we aimed to examine the whole femoral anatomy and gender-specific differences of our study population with 3D measurement methods and to compare our measurement results to the literature.

The high level of accuracy and reliability of the 3D imaging methods for the evaluation of the complex anatomical regions compared to 2D methods were emphasized in many studies in the literature [2,9]. Due to the complex and irregular anatomy of the proximal and distal femur and the need to visualize the whole femoral anatomy for many angle measurements, we have chosen to use whole femoral 3D anatomical models.

The effects of age on anatomical features were determined with the investigation of the different age groups in the literature [3,4,13]. In a study of Buller et al, a negative correlation was found between age and FNI [13]. Schmutz et al. have shown a statistically significant relationship between age and FNA angles [4]. In contrast to these study results, we observed no statistically significant correlation in terms of age - FNA (r = -0.082, p = 0.53) and age - FNI (r = -0.098, p = 0.45). In our study, we investigated the healthy adults in a narrow age range (31-65 years of age) with a mean age of 50.6 ± 8.5 to minimize the effects of age-related anatomical changes on measurement results. The main reason for obtaining the different results from the studies in the literature may be that we analyzed a younger population in a narrow age range.

There is a wide variation about the anatomical landmarks and measurement methods, which are described for the 2D and 3D measurement methods. In our study, we preferred to use some of these predefined landmarks and measurement methods on 3D anatomical models [3,5,6,12]. Despite several methods were identified in the literature for the determination of the femoral neck center, there is no common consensus about the ideal determining method [2,5,11,14]. The femoral neck has a conic shaped missing its top part virtually, although the real femoral neck borders are not known certainly. In some of these studies, the measurement axis and the methods to determine the central point of the neck is not clearly identified [1,3,10,11,14]. Therefore, we marked the narrowest diameter of the femoral neck on 3D models to create a sphere, which fits the surfaces of the bone in all reference axis (XY, XZ and YZ axis). In a study by Park et al., similar marking methods have been used to determine the femoral neck center. In contrast to our study, the authors have created a midline, not a sphere, for the femoral neck measurements [9].

In the current literature, there is no study about the 3D measurement methods, which determines the superiority of a method on the others. Similar to the methods for the determination of the femoral neck center, also different methods were defined to determine the anatomical axis of the femoral shaft in the literature [5,6,8,13,14]. Due to the complex and irregular anatomy of the proximal and distal femur, we decided to create two centrally located spheres on 1/3 proximal and distal femoral borders. This method provides a central line fits to all reference axis on the 1/3 middle part of the femoral shaft, which connects the central points of these spheres.

Anatomical angles are important in the preoperative planning of deformity, arthroplasty or trauma surgeries and for the development of more compatible implants for orthopedic interventions. The comparison of the gender groups in terms of 3D femoral anatomical measurements revealed that a statistically significant difference only in the mean FNA angles (p =0.009). The mean FNA angle in the female group was 17.1 ± 9.9 and in male group was 10.6 ± 8.3. Our study results support the current literature in terms of gender-specific differences of FNA [3,7]. FNA is important for the reconstruction of native hip biomechanics in hip joint arthroplasty procedures. Inappropriate restoration of the FNA and FNI angles in hip arthroplasties cause prosthesis dislocations, impingement and mechanical complications [15,16]. Besides, the retroversion of the femoral neck was detected in twelve adults (six females and six males) in our study. There is only a study in the current literature, that investigate the retroversion ratios of the femoral neck using 3D models in the Caucasian population [17]. The incidence of retroversion was found as 7,8%; however, this study consists of a mixed adult population from different races as Caucasian, Asian, African and Middle East populations [17]. To the best of our knowledge, our study is the first study, that determined the gender-specific retroversion ratios of the Caucasian population using 3D femoral models.

In our study, we measured femoral neck inclination (FNI), femoral mechanical-anatomical axis (FMAA), anatomical lateral distal femoral (aLDFA), mechanical lateral distal femoral (mLDFA), medial distal femoral (MPFA) and lateral proximal femoral (LPFA) angles of Turkish population using 3D computer-based anatomical models (Table 3). Although these angles have shown no statistically significant difference between gender groups of our study, we compared our results with the gender-specific measurement results, that were investigated within the similar age groups in the literature [3,7] (Table 4).

**Table 4:** The comparison of our study results with the literature. FNI: femoral neck inclination; FNA: femoral neck anteversion; mLDFA: mechanical lateral distal femoral angle; MPFA: medial proximal femoral angle; LPFA: lateral proximal femoral angle.

	Our Results		Kulkarni et al.		Degen et al.	
	Female	Male	Female	Male	Female	Male
FNI	125.1	123.1	125.7	128.2	134	132.8
FNA	17.1	10.6	13.2	7.8	18.2	13.2
mLDFA	86.8	86.3	N/A	N/A	86.4	87.8
MPFA	80.9	80.8	N/A	N/A	85.2	85.7
LPFA	92.8	93.1	N/A	N/A	88.6	88.2

In a study by Bozkurt et al., that investigate the proximal femoral anatomy of the Turkish population, the mean FNI angles were found higher than our study results [1]. Although a population of the same origin was examined in this study, taking the reference anatomical axis of proximal femur and the examination of a younger cohort in this study may have yielded different results. The mean gender-specific FNI results of our study were similar with the results of Kulkarni et al., that investigated the anatomical angles of Chinese and South East Asian population [7]. Although the study results of Degen et al., which investigated the Caucasian population similarly, have revealed that the higher mean FNI angles in both gender groups compared to our study results [3]. These differences could be due to the inclusion of younger patients or the investigation of the Caucasian population from western countries in the study of Degen et al [3].

In our study, we determined the higher mean FNA angles compared to Kulkarni et al [7]. Besides, our mean FNA angles were lower than the results of Degen et al [3]. The lower mean MPFA and the higher mean LPFA angles were found in our study compared to Degen et al [3]. We think that the higher FNI angles in the study of Degen et al. could be the main reason for these differences. The higher FNI angles indicate that the more superior placement of the central point of the femoral head sphere in XZ axis. As a result, the line connecting the TrMHead and the central point of the femoral head, which is used for the measurement of the MPFA and LPFA, becomes more vertical in XZ axis.

These anatomical measurements could be used for the preoperative planning in deformity surgeries to obtain a suitable correction by mimicking the native femoral anatomy. Similarly, the anatomical measurements are important details in performing the accurate bone osteotomies, that provide a proper alignment of the extremities for the reconstruction of the native joint biomechanics. Another important point is the development of more compatible implants, prosthesis or biomaterials that could be adapted to larger populations through anatomical data. We think that 3D imaging is a good alternative compared to other imaging techniques to evaluate the real anatomical features accurately. The main purpose of orthopedic implant companies is to develop a best fit implant that can be used for more people with different genders and races [4,7]. The current anatomical data in the literature show that it is still not possible to manufacture a best fit implant for different race and genders [7]. Therefore, the implant manufacturers should offer different designs or implant options, that fit better to the native anatomical differences. Besides, we believe that anatomical data obtained from 3D models could be used for the production of custom-made implants and the printing of 3D anatomical training materials.

There are some limitations in our study. The first limitation could be the total sample size of our study. Due to the inclusion and exclusion criteria of our study, we investigated the right femurs of 60 female and 60 male healthy adults. Total sample size may be considered relatively small, even though the power of the study was found to be 99%. Another limitation of our study includes that all the 3D measurements were performed by a single investigator, who has experience about MIMICS and 3-Matic program and 3D anatomical modeling. However, in our study, the use of predefined points in the literature and the computer-aided automatized methods minimize the measurement errors. Owing to the current study includes only local patients, who administered to our university hospital, it was not possible to compare our 3D measurement results with different races. Therefore, we compared our study results to the different studies in the literature, which have evaluated the gender-related differences of anatomical features with similar measurement methods.

## Conclusions

In conclusion, we determined only a statistically significant difference in femoral neck anteversion (FNA) angles between female and male groups in the Turkish population. Besides, we observed different anatomical measurement results in the Turkish population when compared to other studies in the literature, which investigate the Chinese, South-East Asian or Caucasian population. Through 3D anatomical data, more compatible implants, prosthesis or biomaterials can be manufactured by determining gender and race-specific anatomical differences. We recommend more studies that investigating the effects of implants, that were manufactured in accordance with 3D anatomical data, on the results of orthopedic surgeries.
